# Pyæmia the Result of Alveolar Abscess

**Published:** 1875-12

**Authors:** W. T. Crutchfield


### ARTICLE II.

### *Pyœmia the Result of Alveola Abscess.*

BY W. T. CRUTCHFIELD, M. D.

The journals contain numerous wood cuts presented for the purpose of showing the effects of gum-boil or technically speaking alveolar abscess, and illustrating articles depicting the evils attending this apparently simple trouble—such for instance as deformity of the face, inability to masticate, defective sight, loss of teeth, and such like phenomena; but these I regard as minor points when we consider what may be the final result of such a disease. What accoucher ever goes to the bedside of a lying-in woman who does not apprehend some danger besides that of parturition? If he be a well versed obstetrician he is aware of the dangerous sequelae attending such cases, and is very particular, (however simple the operation may be) to ward off all complications. Now, to be as laconic as possible, let us glance at that part of the science that we are most interested in, oral surgery, and observe the dentist under similar circumstances: A patient presents himself complaining of odontalgia, say of a molar. The diagnosis may show that there is pus around the margin of the gum in the alveoli or an exposed nerve. If there be pus around the margin of the gum, an incision is made,; if in the antrum of highmore, the antrum is perforated by extracting the tooth and carrying the instrument into the gomphosis through the parietes of the maxillary sinus, thereby allowing the pus to exude. If the nerve be exposed, and no destructive inflammation present, the practitioner actuated only by his own judgment, (regardless of what the patient may desire,) will at once cap the nerve with some anodyne, and lay as it were the foundation for filling. But how often it is that the dentist will put his reputation in jeopardy to gratify the whims of irritable people; for fear of displeasing them he will destroy the nerve, thereby leaving a dead tooth to act as a foreign body to the alveoli. The next step may be to fill the tooth which is generally accomplished with the aid of an assistant, who, as the gold is being inserted hammers away with a two ounce mallet, striking about a half pound blow each time. Now there is no doubt in my mind, that this force not only causes irritation, but in many cases periostitis which combined with the efforts of nature to throw off the dead tooth, (as the tendency of nature is to throw off all foreign bodies,) sets up no little inflammation The result may be suppuration and great suffering, and in this manner the deformities we have alluded to may be caused. But let us go deeper into the subject. According to some of the most scientific physiologists, we learn that pus corpuscles enter the circulation. Tthere is no doubt in my humble opinion but that this theory is correct, and from such a cause the state most dreaded by the surgeon—that of pyæmia is induced. This, however, is by no means always the sequence of events, yet 'tis well to ward against a disease that has baffled the skill of the most eminent of the medical profession and the prognosis of which may be said to be that of death, ninety-five per cent. dying.

---

